# Factors Associated with In-Hospital Mortality in Acute Care Hospital Settings: A Prospective Observational Study

**DOI:** 10.3390/ijerph17217951

**Published:** 2020-10-29

**Authors:** Ana María Porcel-Gálvez, Sergio Barrientos-Trigo, Eugenia Gil-García, Olivia Aguilera-Castillo, Antonio Juan Pérez-Fernández, Elena Fernández-García

**Affiliations:** 1 Faculty of Nursing, Physiotherapy and Podiatry, Universidad de Sevilla, 41009 Seville, Spain; aporcel@us.es (A.M.P.-G.); egil@us.es (E.G.-G.); efernandez23@us.es (E.F.-G.); 2 Research Group under the Andalusian Research, Development and Innovation Scheme CTS-1019 Complex Care, Chronic and Health Outcomes, Universidad de Sevilla, 41009 Seville, Spain; 3 Hospital San Agustín de Linares, Servicio Andaluz de Salud, 23700 Linares (Jaen), Spain; oliviaaguilera7@gmail.com; 4 Hospital Santa Ana de Motril Hospital, Servicio Andaluz de Salud, 18600 Motril (Granada), Spain; ajpf76@gmail.com

**Keywords:** hospital mortality, nursing care dependency, nursing staffing level, observational study, multivariate analysis

## Abstract

*Background:* In-hospital mortality is a key indicator of the quality of care. Studies so far have demonstrated the influence of patient and hospital-related factors on in-hospital mortality. Currently, new variables, such as nursing workload or the level of dependency, are being incorporated. We aimed to identify which individual, clinical and hospital characteristics are related to hospital mortality. *Methods*: A multicentre prospective observational study design was used. Sampling was conducted between February 2015 and October 2017. Patients over 16 years, admitted to medical or surgical units at 11 public hospitals in Andalusia (Spain), with a foreseeable stay of at least 48 h were included. Multivariate regression analyses were performed to analyse the data. *Results*: The sample consisted of 3821 assessments conducted in 1004 patients. The mean profile was that of a male (52%), mean age of 64.5 years old, admitted to a medical unit (56.5%), with an informal caregiver (60%). In-hospital mortality was 4%. The INICIARE (Inventario del Nivel de Cuidados Mediante Indicadores de Clasificación de Resultados de Enfermería) scale yielded an adjusted odds ratio [AOR] of 0.987 (95% confidence interval [CI]: 0.97–0.99) and the nurse staffing level (NSL) yielded an AOR of 1.197 (95% CI: 1.02–1.4). *Conclusion*: Nursing care dependency measured by INICIARE and nurse staffing level was associated with in-hospital mortality.

## 1. Introduction

The International Classification for Patient Safety defined an adverse event (AE) as any healthcare-related incident that causes harm to patients [1]. The prevalence of in-hospital AEs in the European Union ranges from 8% to 12%, where 1 in every 100,000 inhabitants dies from this cause, i.e., about 5000 deaths per year [2,3]. The latest report by the Ibero-American Study of Adverse Events (IBEAS) established a prevalence of AEs of 10.5% and a cumulative incidence close to 20% [4]. AEs increase hospital stays by a mean of 8.7 days and double inpatient mortality (relative risk (RR) = 2.10) [5,6].

In-hospital mortality is a key indicator of quality of care that is associated with high-level process indicators [7]. The prevalence of in-hospital mortality is around 5% [8,9]. In addition, there exists an estimated increase mortality of 10% due to unsafe care [10].

In addition, comorbidity also plays an important role, affecting up to 55% of in-hospital deaths [11]. Examples include frailty (adjusted hazard risk (HR) = 4.97) [12], dementia (13.3%) [13] and adverse events such as inpatient fall (18.8%) [14], or inpatient hip fracture (HR = 2.12) in hospitals [15].

Numerous studies propose different explanatory models of hospital mortality [16,17,18,19]. These studies show that in-hospital mortality is influenced by numerous different factors and should be assessed with due consideration of a wide range of potential confounds including patient’s factors (individual and clinical) and hospital-related factors [20].

Age and sex are identified as important factors when considering models of in-hospital mortality. For example, for each year of age, the probability of dying increases by 3% [21]. In fact, the level of dependency is one of the individual factors most frequently associated with negative health outcomes in hospitalised patients [22]. So far, studies have shown the relationship between the level of dependency and mortality, measured either using the Barthel index [23] or the number of nursing diagnoses [24].

However, nursing care dependency may be defined as the care needs that patients have during their hospital stay. In turn, patient care needs result in nursing tasks that increase the workload of nurses. In this context, our research group has developed and validated the INICIARE (Inventario del Nivel de Cuidados mediante Indicadores de Clasificación de Resultados de Enfermería) scale to measure the level of nursing care dependency in hospitalised patients. As a result, INICIARE may also be considered to be a scale for measuring nursing workload, pioneering an approach based on outcomes rather than on interventions or activities [25,26,27].

Finally, among the hospital-related factors, nurse staffing stands out, as numerous studies show that a good nurse staffing may reduce hospital mortality by 12–14% [28,29,30]. Among the clinical factors, an increase in the number of days since admission stands out, as for each additional day since admission the probability of dying increases by 3% [31]. Therefore, the aim of this study was to identify which patient (individual and clinical) and hospital- factors are related to hospital mortality.

## 2. Materials and Methods

### 2.1. Study Design

This was a multicentre prospective observational cohort study.

### 2.2. Participants and Sampling

The present study was conducted between February 2015 and October 2017 in the Andalusian Health Care System (southern Spain), which comprises 26 public hospitals providing health coverage to 8 million citizens. Hospitals were classified in three hospital categories according to their level of specialisation and reference population: primary (>500 beds and large metropolitan areas), specialist (between 200 and 500 beds and small metropolitan areas), and tertiary hospitals (<200 beds and rural areas) [32]. These hospitals represent the specialised healthcare centres of 3.5 million citizens.

The necessary sample size was calculated based on the prevalence of in-hospital mortality, which was 4.4% according to a report by the Spanish National Study on Hospitalisation-Related Adverse Events [33]. By establishing a 99% confidence level and 2% accuracy, the sample amounted to 696 subjects. Stratified consecutive sampling was performed based on (1) hospital size (number of beds), (2) unit (medical or surgical), and (3) sex and age group. A randomisation criterion based on the patient identification number in the electronic health record was included. The following inclusion criteria were used: (1) patients over 16 years of age, (2) admitted to a medical or surgical unit, (3) with a foreseeable stay of more than 48 h.

### 2.3. Data Collection

The inpatients were assessed with the survey by the Registered Nurses (RNs) during their shift. Patients were assessed on the day of their admission and every 48 h subsequently during their hospital stay until discharge or death. The nurses coordinated weekly to review how data were being collected from each of the patients in their units.

The number of participating nurses varied according to the size of the hospital and the number of participating units, from 3–4 nurses in small hospitals to 30–35 in large hospitals. In each hospital, there were two nurses coordinated with the team at their hospital. A training workshop was held for all hospital participants to ensure the proper use of data collection methods and instruments. The RNs participating in the project attended these workshops. In total, 157 registered nurses participated voluntarily and were team members of the funded research project.

The nurse’s coordinator recorded the data collected by the nurses on the encrypted web platform Limesurvey to ensure the correct processing of the data. This process was assessed on a monthly basis during the data collection periods.

### 2.4. Study Variables

The outcome variable studied was the crude mortality rate as an adverse event. The crude mortality rate for a hospital considers the number of patient deaths that occur in a given year in a hospital and then compares it with the number of people admitted for care to that hospital during an equal period.

The independent variables have been grouped into patient (individual and clinical) and hospital factors:

*Individual factors*: Gender (male, female), age and level of education (no education, minimally literate, secondary education, university education).

*Clinical factors*: Length of stay, season (winter, spring, summer, autumn), and other clinical variables, such as cognitive status measured by Pfeiffer’s test, risk of developing pressure ulcers as measured by the INTEGRARE scale (at risk of pressure ulcers, not at risk of pressure ulcers), and level of nursing care dependency as measured by the INICIARE scale, which are detailed below:

Pfeiffer’s test is widely used to assess cognitive status. It consists of 10 items and has two cut-off points. Cognitive impairment is suspected when the error score is equal to 3 or more in people who can read and write, or to 4 or more in people who cannot [34].

The INTEGRARE scale was used to assess pressure ulcer (PU) risk in inpatients. It is a recently created scale with excellent psychometric properties (internal consistency total Cronbach’s α = 0.86). It consists of 6 items measured on a five-point Likert scale (5 reflects the most desirable patient’s condition, and 1 reflects the least desirable). The scoring range is 6–30. Confirmatory factor analysis demonstrated the unidimensionality of the scale, indicating a good model fit (minimum discrepancy per degree of freedom (CMIN/DF) = 4; goodness-of-fit index (GFI), comparative fit index (CFI), normed fit index (NFI), incremental fit index (IFI) = 0.999; root mean squared error approximation (RMSEA) = 0.028) [35].

The INICIARE scale was used to assess nursing care dependency in inpatients [21]. INICIARE is a recently created scale with excellent psychometric properties (intraclass correlation coefficient = 0.830–0.964; overall internal consistency as measured by Cronbach’s α = 0.98, internal consistency of individual subscales as measured by Cronbach’s α ranging between α = 0.92–0.98). It consists of 55 items measured on a five-point Likert scale (5 reflects the most desirable condition of the patient, and 1 reflects the least desirable). The scoring range is 55–275, with three cut-off points (four intervals) that indicate levels of dependency. The seven factors identified using exploratory factor analysis explained 76.8% of the overall variance [36].

All of these scales were administered by registered nurses during their shift.

Hospital-related factors: admission hospital (primary care hospitals, specialty hospitals, and tertiary-care hospitals), type of admission unit (medical, surgical) and nurse staffing level (NSL).

The nurse staffing level is an indicator that represents the available nursing workforce in a hospital. These data were self-reported by the nurses. The nurses’ coordinator reported the number of patients who were admitted to the unit, and the total number of nurses who were scheduled on each shift. Each admission time was recorded considering the patient details and unit. The NSL was calculated as the division of the total number of patients, by the number of experienced nurses that were available on each shift per unit, at each participating hospital [23,37].

### 2.5. Data Analyses

The chi-square statistic was used to test for association between pairs of categorical and ordinal variables. For quantitative variables, non-parametric tests were used (the Mann–Whitney U test and the Kruskall–Wallis test).

A multivariate logistic regression model was conducted in which in-hospital mortality was established as the dependent variable and INICIARE and other related variables (sex, age, length of stay, unit, type of hospital, season, and NSL) were established as independent variables. The calibration and the discrimination of the model were checked using the Hosmer–Lemeshow test and a receiver operating characteristic (ROC) curve, respectively. The data were analysed using the SPSS v. 23 statistical package (SPSS/IBM, Chicago, IL, USA) and R Commander.

### 2.6. Ethical Considerations

This project has been approved by the ethics committee of the Andalusian Healthcare System (Approved 22 March 2013; Code: CPMP/ICH/135/95). Patients were informed verbally and in writing about the objective and purpose of the study. Patients signed an informed consent form and were aware of their right to withdraw from the study at any time. The anonymity of the patients was preserved at all times by using participant numbers. The participating nurses whom collected the data signed a confidentiality agreement consent and were aware of their right to withdraw from the study at any time.

## 3. Results

### 3.1. Sample Characteristics

The sample consisted of 3821 assessments conducted in 1004 patients. The number of assessments ranged from 2 to 28, with a mean value of 3.78 (SD = 2.37). Most of the patients were admitted to medical units (56.3%). The mean profile was that of a male (52%), with a mean age of 64.5 years old, with a caregiver (60%). In-hospital mortality was 4%. Complete patient characteristics can be found in Table 1.

### 3.2. Relationships between Individual, Clinical, Hospital Characteristcs and Hospital Mortality

Table 2 shows the factors related to mortality. Only nursing care dependency, as measured by INICIARE, and NSL were significant variables in the model, with AORs of 0.987 and 1.197, respectively. Other associated factors were being older, being male, staying longer in hospital, being admitted to a medical unit, being admitted to a secondary-care hospital, and being admitted in winter. The model obtained a good adjustment with optimal calibration (Hosmer–Lemeshow: *p* = 0.786) and discrimination (area under curve [AUC] = 0.83 [0.75, 0.90]).

## 4. Discussion

The aim of this study was to identify which patient (individual and clinical) and hospital- characteristics are related to hospital mortality. This is the first study so far to include nursing care dependency of inpatients measured with a validated tool. Each point that the mean of the scale increases corresponds to odds of 0.98 of in-hospital mortality (i.e., the chance of in-hospital mortality decreases by 2%).

Age is one of the main determinants of patient mortality from injuries [38]. Our data indicate that, for each year of life, the probability of dying increases by 1.4%. Patients who died were on average 9 years older than those who lived. The mean age of the deceased (73 years) is similar to that reported in other studies [39]. The ageing of the population is increasing the mean age of hospitalized patients, leading to an increase in mortality and other adverse events, such as medication errors or infections [40,41]. This effect may be greater in medical units, as the mean age is higher in these units than in surgical units [38].

Another variable that influences mortality is the unit of admission, as patients in surgical units are 47.4% less likely to die than patients admitted to medical units. Other studies also showed higher mortality rates in medical units (31.6%) compared to surgical units (10.3%) [42], although surgical units have a higher probability of preventable deaths than medical units (30.8% vs. 13.3) [40].

In relation to seasonality, winter is the season with the highest in-hospital mortality rates. Some studies indicate that winter increases the number of admissions and readmissions of patients with serious diseases, such as heart failure (17.7%), myocardial infarction (6.3%), or pneumonia (51.3%) [43,44], resulting in increased mortality rates of admitted patients compared to other seasons (31.3%) [45,46]. For instance, in the United States, more than 1.5 million adults are admitted for community-acquired pneumonia (CAP) in winter, with 1 in 3 dying during their hospital stay [47].

Finally, among the organisational factors, NSL is directly related to in-hospital mortality. The mean NSL in our study was 10.3, which meant that for each additional patient, in-hospital mortality increased by 19%. A meta-analysis placed this percentage at 14% on average [11] based on studies with values ranging from 5% [48] to 31% [49]. The RN4CAST study found that the mean nurse staffing in European hospitals was 8.3, with Spain being the country with the highest level of nurse staffing (12.7) [21,28]. Our study is consistent with these data and provides evidence of the joint influence of NSL and nursing care dependency on the occurrence of in-hospital mortality. Future studies should incorporate this measure into the adjustment of the nurse staffing level to decrease in-hospital mortality.

Some limitations can be highlighted in this study. Not all relevant variables that have been demonstrated to affect patient mortality have been included here. For example, the literature has shown that patients with a higher probability of dying have a higher number of comorbidities [11,12], frailty [13], and dementia [14]. In our study we have included the evaluation of the cognitive state [34], which also affects mortality; nonetheless, it was not an explanatory variable in our model. Although hospital adverse events have also been shown to have a relationship with hospital mortality [33,39], all of them were not included. Our study has included a measure of the risk of ulcer pressure, a hospital adverse event. Future studies could include these variables to adjust for NSL and the effect of dependency on nursing care on hospital mortality.

## 5. Conclusions

Several factors related to hospital mortality have been investigated in the current study: (1) individual factors such as sex and age; (2) clinical factors such as length of stay, season, and nursing care dependency (measured by INICIARE9) and (3) hospital factors such as admission hospital, unit and NSL.

Nursing care dependency measured by INICIARE and NSL was the only significantly associated factor with in-hospital mortality. Including this scale in the assessment of patients both upon admission and during their hospital stay would improve models of in-hospital mortality and allow the level of patient care in hospitals to be measured and quantified.

The finding in this study have important research applications. On one hand, it would enable the setting up of more complex and precise models that explain hospital mortality. On the other hand, it also shows how the nursing care influences hospital mortality: both in terms of the number of nurses (NSL) and the amount of nursing care provided (INICIARE).

In conclusion, the most important implications for health care policy-makers should be aimed at controlling the hospital factors that influence hospital mortality. The results of this study show that factors such as the type of hospital and the type of unit determine the death of the patient. Future studies should involve an in-depth analysis of patient care in these settings. Among the hospital factors, NSL stands out, showing a statistically significant relationship with hospital mortality. Health care policy-makers should study the status of their NSL at different levels (hospital and unit) in order to improve the provision of nursing staff and to provide safe and quality care.

## Figures and Tables

**Table 1:** Sample characteristics.

	All Patients (*n* = 1004)	Dead Patients (*n* = 40)	Living Patients (*n* = 964)	Dead Patients vs. Living Patients
**Individual’s factors**				
Sex				0.717
Female	480 (47.8)	18 (45)	462 (47.8)	
Male	524 (52.2)	22 (55)	502 (52.2)	
Age				0.001
Mean (SD)	64.5 (17.1)	73.3 (12.7)	64.2 (17.2)	
Level of education				0.008
No education	229 (22.7)	13 (32.5)	216 (22.5)	
Minimally literate	518 (51.6)	26 (65)	492 (51)	
Secondary education	195 (19.4)	1 (2.5)	194 (20)	
University education	62 (6.2)	0	62 (6.5)	
**Clinical factors**				
Length of stay				0.074
Mean (SD)	10.7 (10.7)	16 (21.7)	10.5 (10)	
Pfeiffer’s test				0.002
Oriented	866 (86.3)	28 (70)	838 (87)	
Other	138 (13.7)	12 (30)	126 (13)	
INTEGRARE At risk of pressure ulcers Not at risk of pressure ulcers				0.035
	486 (48) 518 (52)	26 (65) 14 (35)	460 (48) 504 (52)	
INICIARE mean during stay				<0.001
Mean (SD)	217.5 (43.1)	174.2 (56.6)	219.3 (41.5)	
Season				0.004
Winter	383 (38)	22 (55)	361 (37.4)	
Spring	448 (44.8)	10 (25)	445 (45.6)	
Summer	101 (10)	2 (5)	99 (10.4)	
Autumn	62 (6.2)	6 (15)	56 (6)	
**Hospital factors**				
Admission hospital				0.312
Regional	664 (66)	23 (57.5)	641 (66.5)	
Medical specialties	165 (16.4)	10 (25)	155 (16)	
District	175 (17.4)	7 (17.5)	168 (17.5)	
Unit				0.015
Medical	565 (56.3)	30 (75)	535 (55.5)	
Surgical	439 (43.7)	10 (25)	429 (44.5)	
Nurse staffing level				
Mean (SD)	10.26 (2.74)	10.35 (2.24)	10.26 (2.78)	0.901

**Table 2:** Multivariate logistic regression model.

	*Β*	95% CI	*p*	VIF
(Intercept)	0.388		0.528	
Sex [Male]	Reference			
Sex [Female]	0.811	0.409; 1.611	0.55	1.097154
Age	1.014	0.988; 1.04	0.299	1.323345
Length of stay	1.013	0.98; 1.03	0.275	1.090534
INICIARE centred mean: 217	0.983	0.976; 0.991	<0.001 *	1.302930
Season [Winter]	Reference			
Season [Spring]	0.265	0.116; 0.606	0.002 *	1.101491
Season [Summer]	0.223	0.043; 1.146	0.072	
Season [Autumn]	0.497	0.092; 2.05	0.369	
Hospital [Regional]	Reference			
Hospital [Specialty]	1.338	0.528; 3.389	0.539	1.143941
Hospital [District]	0.829	0.327; 2.099	0.692	
Unit [Medical]	Reference			
Unit [Surgical]	0.526	0.238; 1.162	0.112	1.142948
Nurse Staffing Level	1.197	1.02; 1.4	0.0215*	1.120576

* *p* < 0.05. Model calibration (Hosmer–Lemeshow test): *p* value = 0.7861. Model discrimination (receiver operating characteristic (ROC) curve): area under curve (AUC) = 0.83 (95% confidence interval (CI) [0.75; 0.90]. CI: Confidence Intervals; VIF: Variance Inflation Factor.
